# Remarks on Mr. Gilder's "Case of Vaccine Disease and Measles Existing at the Same Time in the Same Individual."

**Published:** 1822-09

**Authors:** 


ART. X.—*Remarks on* MR. GILDER'S "*Case of Vaccine Disease and Measles existing at the same time in the same Individual.*" By H.*

THE last volume of the Medico-Chirurgical Transactions contains an interesting case of vaccine disease co-existent with measles, which occurred under the observation of Mr. Gilder. Both diseases appeared to run their natural courses, without influencing each other. The anomaly, therefore, is, that the suspension of one or other of them did not take place; a circumstance which, *a priori*, might naturally have been expected. Mr. Gilder presumes, that the case "will go to prove that two diseases, hitherto accounted distinct, may exist, and proceed uninterruptedly, in the same constitution and at the same time, without any apparent deviation from the regularity which each disease would exhibit *per se*." He also thinks that the case subverts the theory of John Hunter, that no two actions can take place in the constitution, or in the same part, at the same time. There, however, appears to me nothing in the case that is irreconcileable to Hunter's doctrine. What proof is there that two simultaneous actions existed in the constitution? It is, certainly, not fair to infer that, because the vaccine vesicles went through their ordinary progress, that therefore the constitution received its ordinary influence. There is no peculiarity in the symptoms which the system sometimes presents under the vaccine disease, and usually they are so slight as to escape cognizance: and certainly if any had existed in this case, they would have been lost in the higher excitement produced by the measles,

* The Editors earnestly request that gentlemen, who favour them with Communications, will have the goodness to give their names. It must be obvious how much the value of an original paper is diminished by its being anonymous.

as much as the areolæ of the vesicles were obscured "by the darker eruption of the measles." The question then is, has the infant lost its susceptibility for small-pox? If it has, then there is some proof of the constitution having been impressed by two simultaneous actions. But I am inclined to believe that the susceptibility for small-pox would be found still to remain, if the experiment of inoculation were to be performed; and it is only by this experiment that proof can be obtained, or truth elicited.

That two local actions did not take place simultaneously in the same part, is pretty evident from the fact of the vaccine vesicles exhibiting their usual progressive phenomena, and from the circumstance of the lymph taken from one of them, for the purpose of vaccination, producing the genuine disease. It would be unphilosophical to suppose that a combination of two different actions would produce the identical effects which are characteristic of, and always flow from, one of the actions. Surely some modification would result from the actions being compounded, and something in the shape of a *tertium quid* be generated.

Mr. Gilder's case is, however, curious, inasmuch as it shows unequivocally that the local effects of vaccination may exist simultaneously with measles. The great Hunter has related a case where the usual progress of inoculation was suspended in consequence of the appearance of measles; on the disappearance of which complaint, the small-pox came forward, and went through its natural course.

"In what the distinction consists, in so far as the constitution is concerned, in regard to its capacity for receiving the infection of the matter of the cow-pox, and not of the small-pox, whilst evidently labouring under a specific disease," Mr. G. does not attempt to discuss; nor shall I attempt it, otherwise than by asking the question, whether there is necessarily connected with the progress of small-pox pustule a degree of constitutional influence? If this be affirmatively answered, then the progress of the pustules may be arrested, because the constitutional excitement is prevented from taking place on account of the system being impressed by a different action. That there is not necessarily a constitutional action attending the progress of vaccine vesicles, is rendered extremely probable by Mr. Gilder's case; and I have reason to believe that many of the failures of vaccination are owing to this want of sympathy or co-operation between the constitution and the local action. It is, therefore, an imperative duty on every practitioner to ascertain well the state of his subject before vaccination is performed, lest any latent constitutional disorder may counteract the beneficial influence of the virus, though not its local effects.

*Chester; July 27th,* 1822.

